# EDEM2 and OS-9 Are Required for ER-Associated Degradation of Non-Glycosylated Sonic Hedgehog

**DOI:** 10.1371/journal.pone.0092164

**Published:** 2014-06-09

**Authors:** Hsiang-Yun Tang, Chih-Hsiang Huang, Ya-Han Zhuang, John C. Christianson, Xin Chen

**Affiliations:** 1 Institute of Biotechnology and Pharmaceutical Research, National Health Research Institutes, Chunan town, Miaoli, Taiwan, ROC; 2 Ludwig Institute for Cancer Research, University of Oxford, ORCRB, Headington, Oxford, United Kingdom; 3 Graduate Institute of Basic Medical Science, China Medical University, Taichung, Taiwan, ROC; University of Pittsburgh, United States of America

## Abstract

Misfolded proteins of the endoplasmic reticulum (ER) are eliminated by the ER-associated degradation (ERAD) in eukaryotes. In *S. cerevisiae,* ER-resident lectins mediate substrate recognition through bipartite signals consisting of an unfolded local structure and the adjacent glycan. Trimming of the glycan is essential for the directional delivery of the substrates. Whether a similar recognition and delivery mechanism exists in mammalian cells is unknown. In this study, we systematically study the function and substrate specificity of known mammalian ER lectins, including EDEM1/2/3, OS-9 and XTP-3B using the recently identified ERAD substrate sonic hedgehog (SHH), a soluble protein carrying a single N-glycan, as well as its nonglycosylated mutant N278A. Efficient ERAD of N278A requires the core processing complex of HRD1, SEL1L and p97, similar to the glycosylated SHH. While EDEM2 was required for ERAD of both glycosylated and non-glycosylated SHHs, EDEM3 was only necessary for glycosylated SHH and EDEM1 was dispensable for both. Degradation of SHH and N278A also required OS-9, but not the related lectin XTP3-B. Robust interaction of both EDEM2 and OS-9 with a non-glycosylated SHH variant indicates that the misfolded polypeptide backbone, rather than a glycan signature, functions as the predominant signal for recognition for ERAD. Notably, SHH-N278A is the first nonglycosylated substrate to require EDEM2 for recognition and targeting for ERAD. EDEM2 also interacts with calnexin and SEL1L, suggesting a potential avenue by which misfolded glycoproteins may be shunted towards SEL1L and ERAD rather than being released into the secretory pathway. Thus, ER lectins participate in the recognition and delivery of misfolded ER substrates differently in mammals, with an underlying mechanism distinct from that of *S. cerevisiae.*

## Introduction

Proteins that misfold in the endoplasmic reticulum (ER) are cleared through a ubiquitin-proteasome dependent mechanism known as ER-associated degradation (ERAD) [1]. Misfolded proteins are initially recognized by ER-resident chaperones and lectins [2] and through pathways that are not entirely delineated, many ERAD substrates are delivered to the membrane-embedded ubiquitin ligase HRD1 (Hrd1p in *S. cerevisiae*). HRD1 interacts directly with SEL1L (Hrd3p in *S. cerevsiae*), an important scaffold protein for HRD1-mediated ERAD [3], [4]. Substrates are then retro-translocated across the ER membrane, ubiquitinated on the cytosolic side of the ER, extracted from the membrane by the AAA-ATPase p97 (cdc48 in *S. cerevisiae*), and degraded by the 26S proteasome.

ER-resident lectins play an essential role in distinguishing terminally misfolded proteins from folding intermediates and how they do so has been the subject of intensive investigation. Based on studies in *S. cerevisiae* using the model substrate CPY*, it is thought that substrate recognition and targeting for ERAD requires a bipartite signal consisting of an unfolded local structure and an adjacent trimmed glycan [5], [6]. In the absence of the glycan, substrates are retained inside the ER instead of being targeted for degradation [5], [7]. The lectins Htm1p and Yos9p are both essential for ERAD in yeast [8], [9], [10]. Htm1p trims substrate’s high mannose oligosaccharides to expose α1,6 mannose moieties [11], [12], [13], which can then be recognized through the mannose-6-phosphate receptor homology (MRH) domain of Yos9p [14], [15], [16]. Yos9p also interacts with Hrd3p, the interaction partner of the ubiquitin ligase Hrd1p [17], [18], thus permitting substrates to be delivered from Yos9p to Hrd1p via Hrd3p [19], [20]. The observation that there is no additive effect on degradation with deletion of both Htm1p and Yos9p (*htm1*Δ/*yos-9*Δ), supports the model of sequential processing of the glycan on substrates by these lectins [21].

Compared to the yeast, the mammalian ERAD system is far more complex and less defined. Three Htm1p (EDEM1, EDEM2 and EDEM3) and two Yos9p orthologs (OS-9 and XTP3-B) have been identified. Similar to Htm1p and Yos9p, EDEM1 and OS-9 appear to discriminate misfolded proteins from native forms to initiate their turnover [22], [23], [24]. For a few ERAD substrates whose degradation requires EDEM1 and OS-9, the trimmed glycan structure on the substrates was found essential for recognition [23], [24], [25]. However, it is not yet known whether in mammalian cells substrate sorting and targeting towards ERAD requires glycan processing, and if so, whether the processing is carried out by these lectins. Intriguingly, with the exception of XTP3-B [26], the lectins EDEM1, EDEM2, EDEM3 and OS-9 do not play any role in the degradation of several unglycosylated ERAD substrates, despite of the facts that they were found in association with these non-glycosylated substrates [8], [23], [24], [27], [28], [29], [30].

We have previously demonstrated that the human sonic hedgehog (SHH) protein is processed by self-cleavage in the ER, generating a N-terminal fragment secreted for signaling, and a C-terminal fragment degraded by HRD1-mediated ERAD [31]. The N-linked oligosaccharide appended to the C-terminal fragment of SHH (SHH-C) in the ER is removed upon retro-translocation to cytosol [31]. To understand the role of N-glycans in substrate recognition, we generated N278A, a non-glycosylated SHH mutant. We showed that N278A fails to fold properly and is targeted for ERAD in a process that requires HRD1, SEL1L and p97. By comparing degradation of SHH-C and N278A, we sought to probe the essential components involved in recognition and processing of substrate glycans and define the role of individual ER lectin in this critical step of the ERAD pathway.

## Results

### N278A is Degraded by HRD1-mediated ERAD

**Figure 1:**
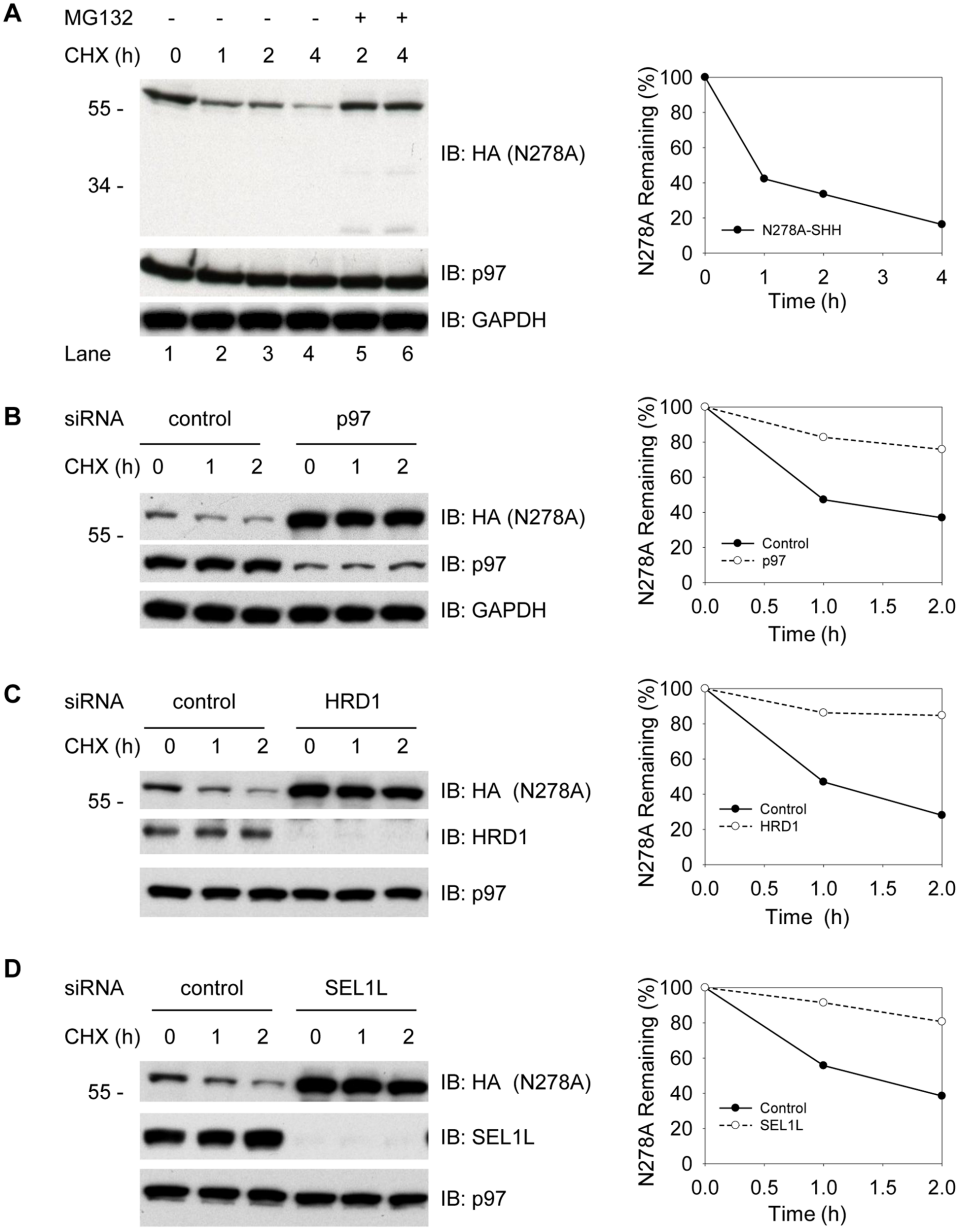
ERAD components required for the degradation of N278A. (**A**) N278A-HA was stably expressed in 293T cells. Protein synthesis was inhibited with cycloheximide (CHX), and the fate of the protein was followed. Samples were analyzed by SDS-PAGE and immunoblotting with HA antibodies. Immunoblotting with p97 and G3PDH were used as loading controls. (**B**) Cells stably expressing N278A-HA were depleted of AAA-ATPase p97, and the fate of N278A proteins was followed after CHX addition. All samples were analyzed by SDS-PAGE and immunoblotting with HA antibodies. The extent of depletion was shown by immunoblotting with p97 antibodies. Controls were treated with commercial negative control siRNA. The right graph shows quantification of N278A in the experiment. Immunoblotting for GAPDH served as loading control. (**C**) As in (**B**), but with depletion of ubiquitin ligase HRD1. Immunoblotting for p97 served as loading control. (**D**) As in (**C**), but with depletion of SEL1L. Molecular masses are given in kilodaltons.

Human SHH is a glycoprotein with a canonical N-linked glycosylation motif (N_278_QS) [31]. To determine whether this glycan is essential for the processing and degradation of SHH, we used site-directed mutagenesis to generate a non-glycosylated variant (N278A). The N278A mutant was unable to undergo auto-cleavage (Figure 1A, lane 1), indicating that the correct folding/processing of SHH requires glycosylation at this conserved site. Treatment of N278A with PNGase confirmed its non-glycosylated state (Figure S1). It is noteworthy that the mutant protein migrated slower than the expected molecular mass. We have confirmed that the slower migration of mutant SHH is caused by the mutation. Next, we carried out cycloheximide chase experiment to determine the half-life of N278A. The non-glycosylated N278A mutant was unstable and degraded with a half-life of less than 1 hour (Figure 1A, lanes 1 to 4). As anticipated, the degradation of SHH N278A was mitigated by addition of the proteasome inhibitor MG132 (Figure 1A, lanes 5 and 6), confirming the proteasome-dependent disposal following presumed retro-translocation from the ER.

To identify factors required for N278A degradation, we used RNAi-mediated gene silencing that targeted a selection of established ERAD components. Depletion of p97, HRD1 and SEL1L almost completely stabilized N278A (Figure 1B to 1D). In addition, expression of a catalytically inactive E3 ubiquitin ligase HRD1 or p97 ATPase mutants also strongly impaired degradation of N278A (Figure S2, A and B). These results demonstrate N278A as a bona fide ERAD substrate that requires p97, HRD1 and SEL1L for its disposal.

### Degradation of Glycosylated SHH is Inhibited by Glucosidase and Mannosidase Inhibitors

**Figure 2:**
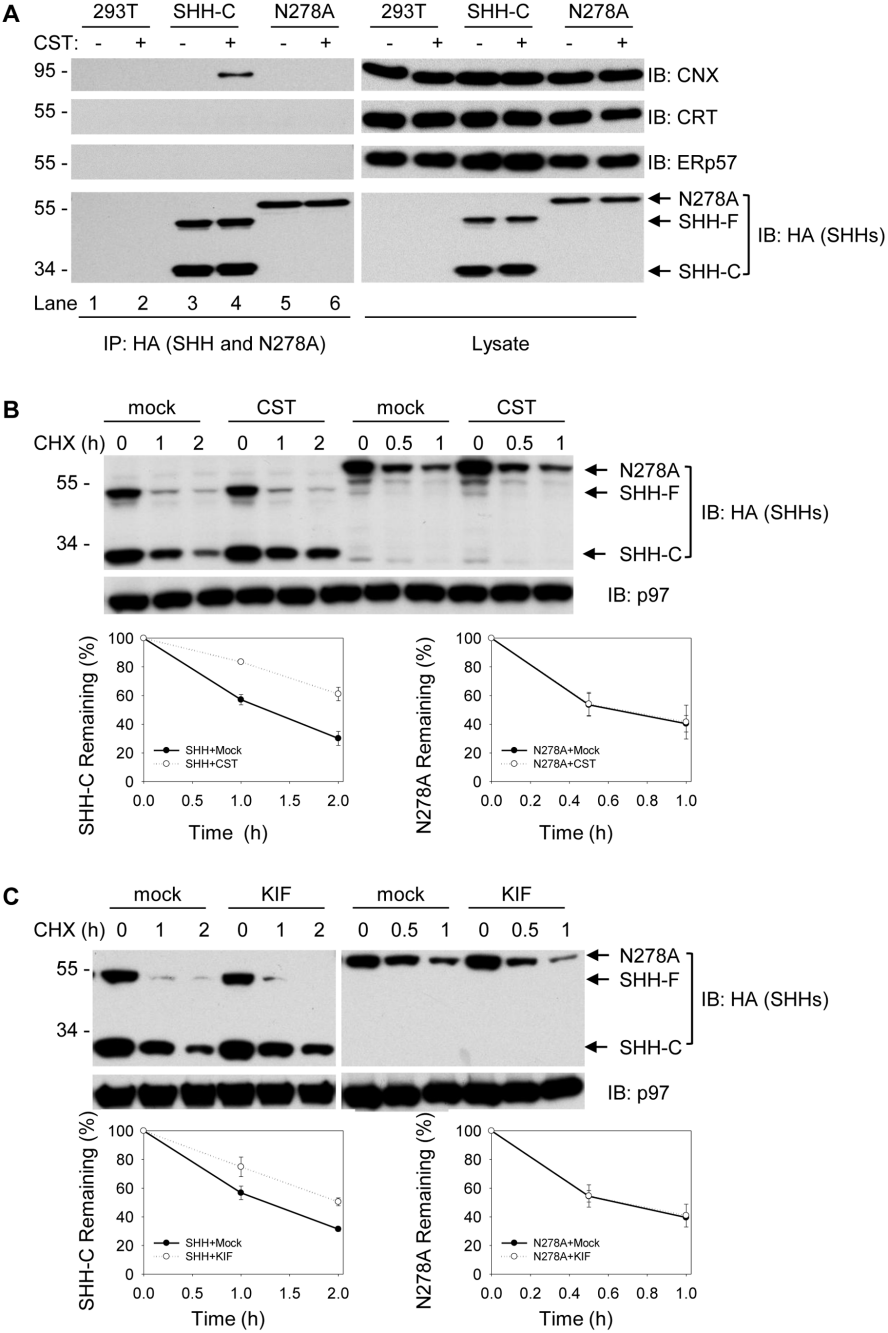
Effects of glucosidase and mannosidase inhibitors on the interaction and degradation of SHH-C and N278A proteins. SHH-F: full length SHH. (**A**) Cells stably expressing either SHH-HA or N278A-HA were lysed in buffer with protease inhibitors and 1% digitonin, and immunoprecipitated with HA antibodies. The precipitates and total cell lysate were separated in SDS-PAGE and immunoblotting with antibodies that recognize the endogenous proteins, including calnexin (CNX), calreticulin (CRT), ERp57, and HA-tagged SHH proteins. Alternatively, the cells were treated with castanospermine (CST) (0.2 mg/ml) for 90 mins before collected for lysis. (**B**) Cells stably expressing either SHH-HA precursor or N278A were treated with CST (0.2 mg/ml) for 90 mins followed by cycloheximide (CHX) addition. The fate of SHH-C or N278A was followed by immunoblotting with HA antibodies. Immunoblotting for p97 served as loading control. The lower graphs show quantification of SHH-C and N278A from three independent experiments. (**C**) as in (**B**), but treated with KIF (0.1 mM).

Within the ER lumen, glucosidase I and II trim the N-linked oligosaccharides of nascent chains to a monoglucosylated form that can be recognized and bound by the ER chaperone/lectin calnexin to promote folding [32]. Inhibition of glucosidases by CST results not only in failure of the glycosylated proteins to enter the calnexin binding cycle, but also in blockage of the release of monoglucosylated proteins from calnexin [32]. To determine whether the non-glycosylated SHH variant must necessarily enter the calnexin folding cycle prior to ERAD, we treated SHH and SHH-N278A expressing cells with glucosidase inhibitor castonospermine (CST) and probed for interaction with calnexin. While glycosylated SHH pulled down calnexin in the presence of CST, N278A did not (Figure 2A, compare lanes 4 and 6). Under the same condition, neither SHH protein was able to precipitate calreticulin or ERp57 (Figure 2A). These results support the conclusion that SHH is folded, at least partially, through calnexin machinery.

Demannosylation by ER mannosidase I permits misfolded proteins to exit the calnexin binding cycle for ERAD [33]. To determine whether glucosidases and ER mannosidase I participate in the degradation of SHH and N278A degradation, we treated the cells with CST and the mannosidase I inhibitor kifunensine (KIF) for 90 minutes prior to cycloheximide chase and monitored substrate degradation. Both CST and KIF inhibited degradation of glycosylated SHH-C (Figures 2B and 2C, lanes 4–6 vs 1–3), but had no effect on clearance kinetics of unglycosylated N278A (Figures 2B and 2C, lanes 10–12 vs 7–9). These results collectively argue that glycosylated SHH is extracted from the calnexin folding system and targeted to the ERAD while the non-glycosylated N278A variant is degraded without encountering calnexin.

### Contribution of EDEM2 and EDEM3 for ERAD of SHH-C and N278A Proteins

**Figure 3:**
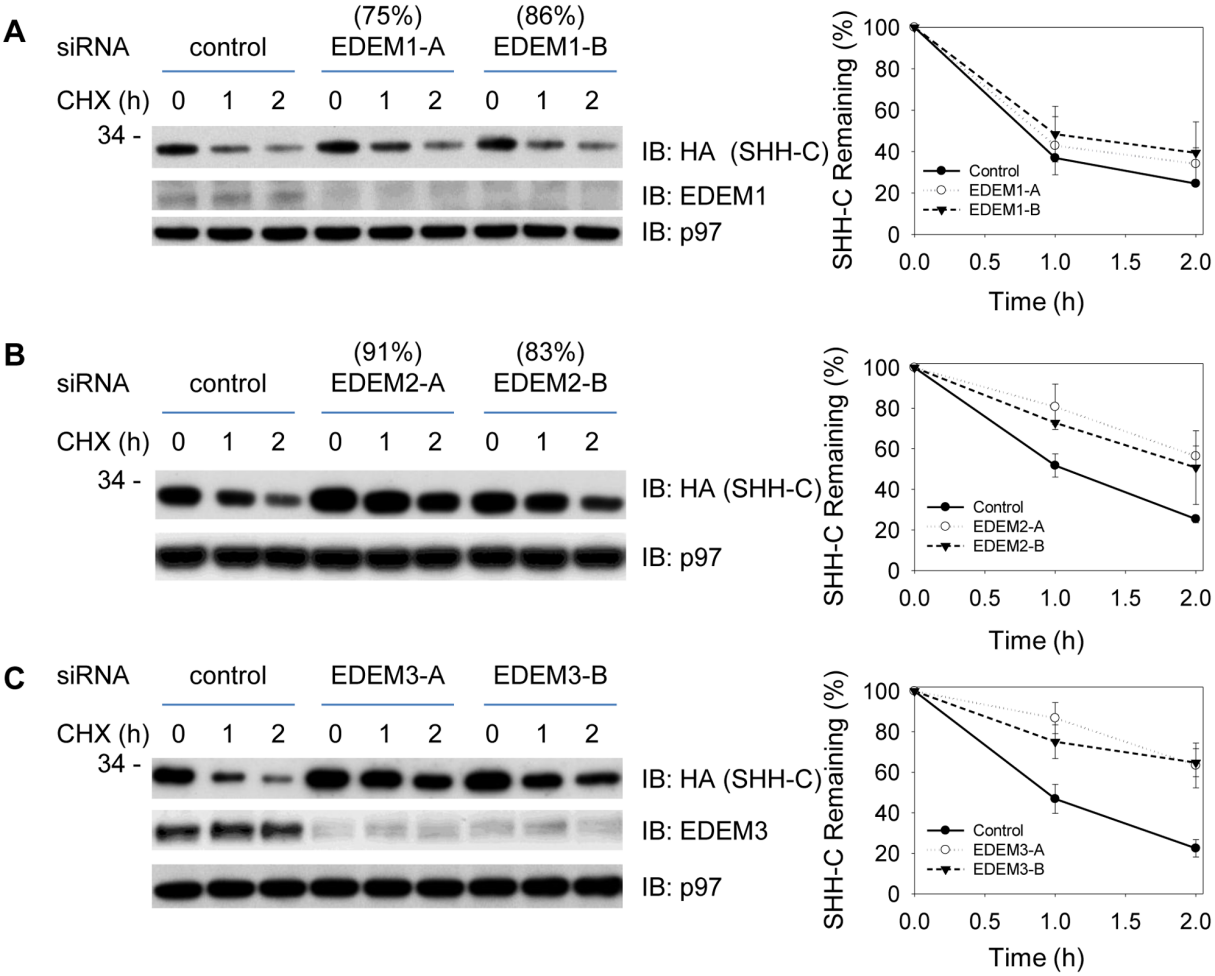
Contribution of EDEMs to the degradation of glycosylated SHH-C proteins. All the experiments have been repeated and one representative blot is shown here. Error bars represent the standard deviations calculated from three independent repeats. (**A**) Cells stably expressing the SHH-HA precursor were depleted of EDEM1 by siRNA, and the fate of SHH-HA was followed after cycloheximide (CHX) addition. All samples were analyzed by SDS-PAGE and immunoblotting with HA antibodies. Controls were treated with a commercial negative control siRNA. The extent of the EDEM1 depletion (in parentheses) was determined by quantitative RT-PCR. The samples were also immunoblotted for EDEM1 to show the extent of depletion. Immunoblotting with p97 antibodies served as loading control. (**B**) As in (**A**), but with depletion of EDEM2 by siRNA. The extent of the EDEM2 depletion (in parentheses) was determined by quantitative RT-PCR. (**C**) As in (**A**), but with depletion of EDEM3 by siRNA. The samples were also immunoblotted for EDEM3 to show the extent of depletion.

**Figure 4:**
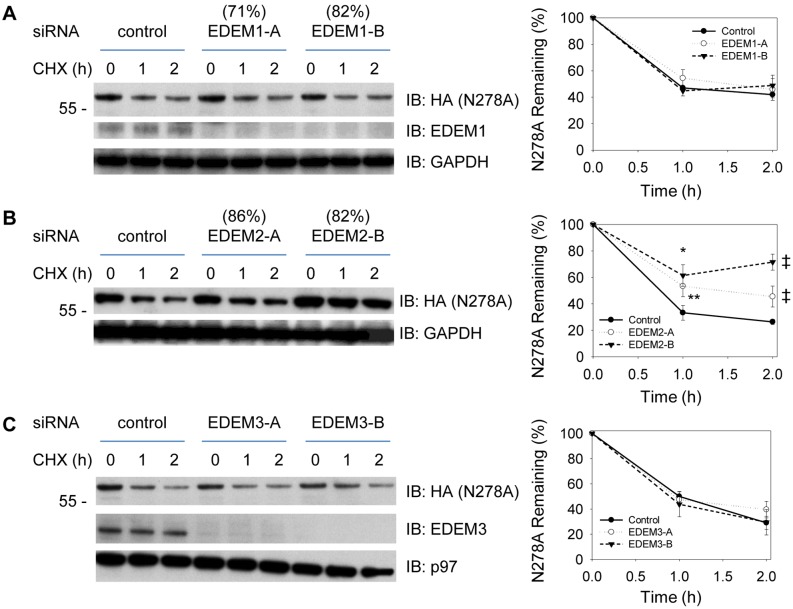
Contribution of EDEMs to the degradation of non-glycosylated N278A proteins. All the experiments have been repeated at least three times and one representative blot is shown here with error bars representing standard deviation calculated from at least three independent repeats. (**A**) Cells stably expressing the N278A-HA precursor were depleted of EDEM1 by siRNA, and the fate of N278A was followed after cycloheximide (CHX) addition. All samples were analyzed by SDS-PAGE and immunoblotting with HA antibodies. Controls were treated with a commercial negative control siRNA. The right graph shows quantification of N278A from three independent experiments. The extent of the EDEM1 depletion (in parentheses) was determined by quantitative RT-PCR. The samples were also immunoblotted for EDEM1 to show the extent of depletion. Immunoblotting with p97 antibodies served as loading control. (**B**) As in (**A**) but with depletion of EDEM2. The extent of the EDEM2 depletion (in parentheses) was determined by quantitative RT-PCR. Standard deviations were calculated from four independent experiments. *: p<0.0001; **: p<0.001; ‡: p<0.00001. (**C**) As in (**A**), but with depletion of EDEM3. The samples were also immunoblotted for EDEM3 to show the extent of depletion.

We next determined the contribution of each EDEM family member to the degradation of SHH-C and N278A using RNAi-mediated gene silencing. Because the antibodies currently available are unable to recognize endogenous EDEM2, We have used quantitative PCR (qPCR) to confirm that each siRNA oligo used in this study reproducibly knocked down its target mRNA. Assessing SHH degradation by CHX-chase assay, we observed that depletion of EDEM1 by two different siRNAs had no effect on SHH-C degradation (Figure 3A), while depletion of EDEM2 or EDEM3 both resulted in significant inhibition (Figure 3B and 3C). When similar experiments were carried out with cells stably expressing SHH N278A, it was noted that N278A degradation was inhibited by depletion of EDEM2, but not EDEM1 or EDEM3 (Figure 4). Thus, EDEM2 appeared to be the only family member required for ERAD of nonglycosylated N278A, while both EDEM2 and EDEM3 were necessary for ERAD of glycosylated SHH-C. It is noteworthy that we observed reproducibly better inhibition with EDEM2-B oligo than EDEM2-A oligo for N278A degradation, even though the two siRNAs reduces the EDEM2 mRNA to a similar extent. Both these oligos have no observable effects on the total amount of the known ERAD components (Data not shown). At the moment, we attribute the difference observed to some unknown factors associated with siRNA experiments in mammalian cells.

### Interaction of EDEMs with SHH and N278A Proteins

**Figure 5:**
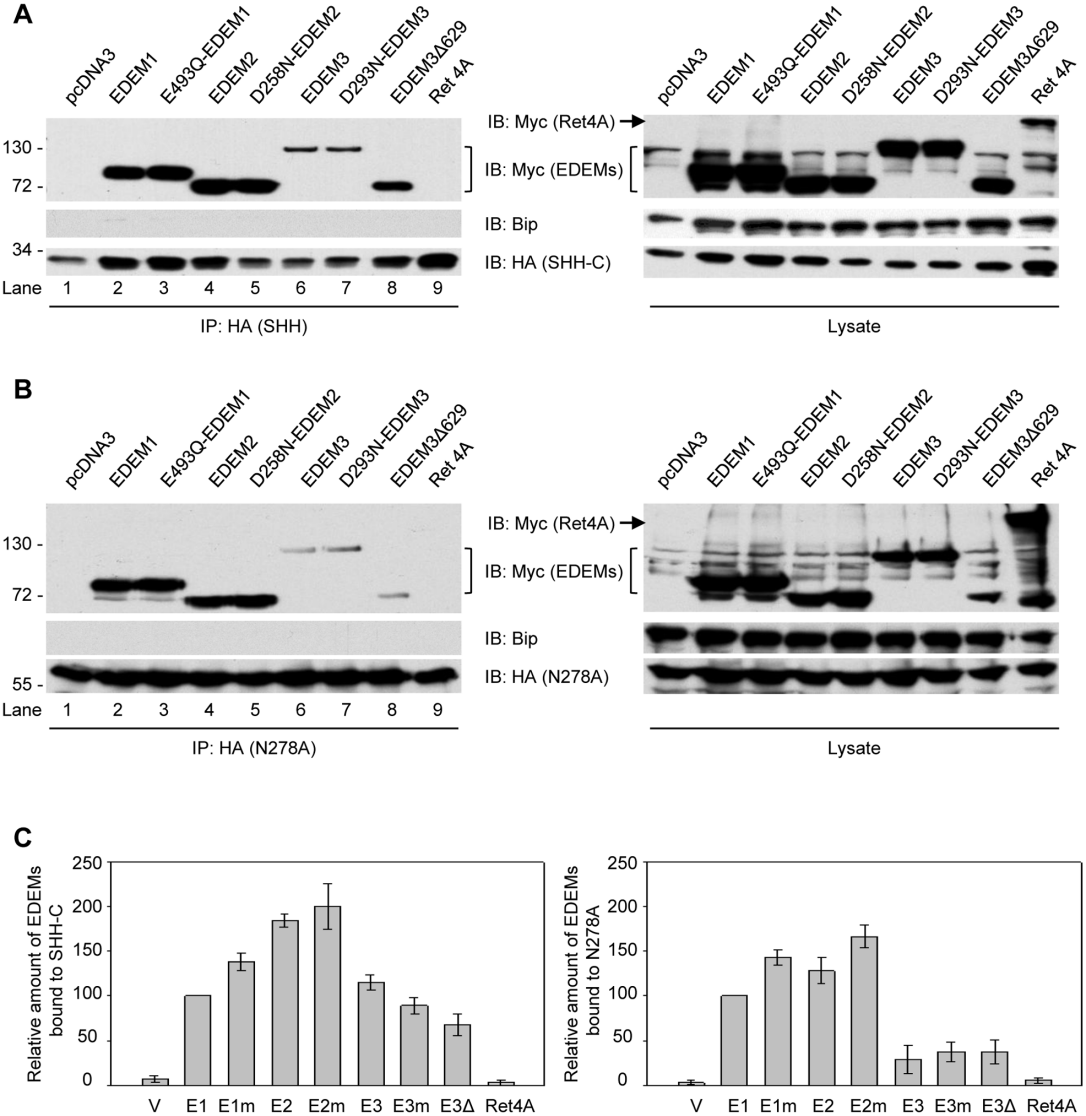
Interaction of SHH proteins with EDEMs. (**A**) Cells were lysed in buffer containing 1% Triton and immunoprecipitated by HA antibodies. The precipitates and total cell lysate were separated in SDS-PAGE and immunoblotting with HA and Myc antibodies. As control, empty plasmid vector or plasmid encoding ER membrane protein Ret4A was transfected. (**B**) As in (**A**), but with N278A-HA plasmids. (**C**) Quantification of the amount of EDEMs bound to SHH-C (left) or N278A (right). Error bars were standard deviations from three independent repeats.

EDEM2 was required for the ERAD of both glycosylated and nonglycosylated SHH (Figures 3 and 4), and so we investigated whether interactions were occurring between EDEM2 and the ERAD substrates. The continuous flow of substrates towards the proteasome by way of ERAD implicitly suggests that any contact between substrates and the ER lectins is likely to be transient in nature, making detection of such interactions potentially problematic. In an attempt to capture such dynamic interactions, plasmids encoding ER lectins and HA-tagged SHH or N278A were transiently co-transfected into cells. To minimize any potential over-expression artifact, we titrated down the amount of plasmid to one-tenth of the normal amount (Materials and Methods). Immunoprecipitations of both SHH-C and N278A were able to bring down all three Myc-tagged wild type EDEMs (Figures 5A and 5B, lanes 2, 4 and 6), suggesting that interactions with EDEMs were independent of the substrate’s glycosylation state. This is consistent with transient and dynamic interactions associated with the delivery and transfer of substrates for degradation by the lectins. Compared to EDEM1 and EDEM2, EDEM3 exhibited the weakest binding towards either glycosylated or nonglycosylated substrates (Figure 5, lane 6 vs 2 and 4). As SHH was unable to co-precipitate the abundant ER membrane protein reticulon 4A (Figure 5A and 5B, lane 9), interactions with EDEM1 and 2 appear to be specific. Unlike EDEM1 and EDEM2, EDEM3 has a C-terminal extension whose function has not been fully ascertained [28]. One possibility may be that this extra domain hinders the interaction with the substrates. Indeed, removing this domain (EDEM3Δ629) increased the interaction between EDEM3 with SHH-C (Figures 5A, lane 8).

To further confirm that EDEM1/2/3 and SHH-C interact through an oligosaccharide-independent mechanism, we introduced mutations to the active site of each EDEMs (EDEM1_E493Q_, EDEM2_D258N_, EDEM3_D293N_) that abolished not only intrinsic mannosidase activity (if present) but also any glycan binding capacity [22]. Interestingly, both SHH-C and N278A co-precipitated with these mutant EDEMs, comparably to their wild type counterparts (Figure 5, lanes 3, 5 and 7). We detected residual binding of EDEM3 for N278A proteins (Figure 5B, lanes 6 and 7). Moreover, deletion of its C-terminal extension did not improve the binding (Figure 5B, lane 8). Quantification of the amount of different EDEMs bound with either SHH-C or N278A were presented in Figure 5C. Taken together, these results indicate that glycan recognition and binding are not required for EDEM1-3 to interact with misfolded polypeptides. Interactions between SHH and EDEMs may be mediated by the recognition of the SHH polypeptide backbone, perhaps through unfolded or exposed hydrophobic regions.

### Contribution of OS-9 to the Degradation of N278A Proteins

**Figure 6:**
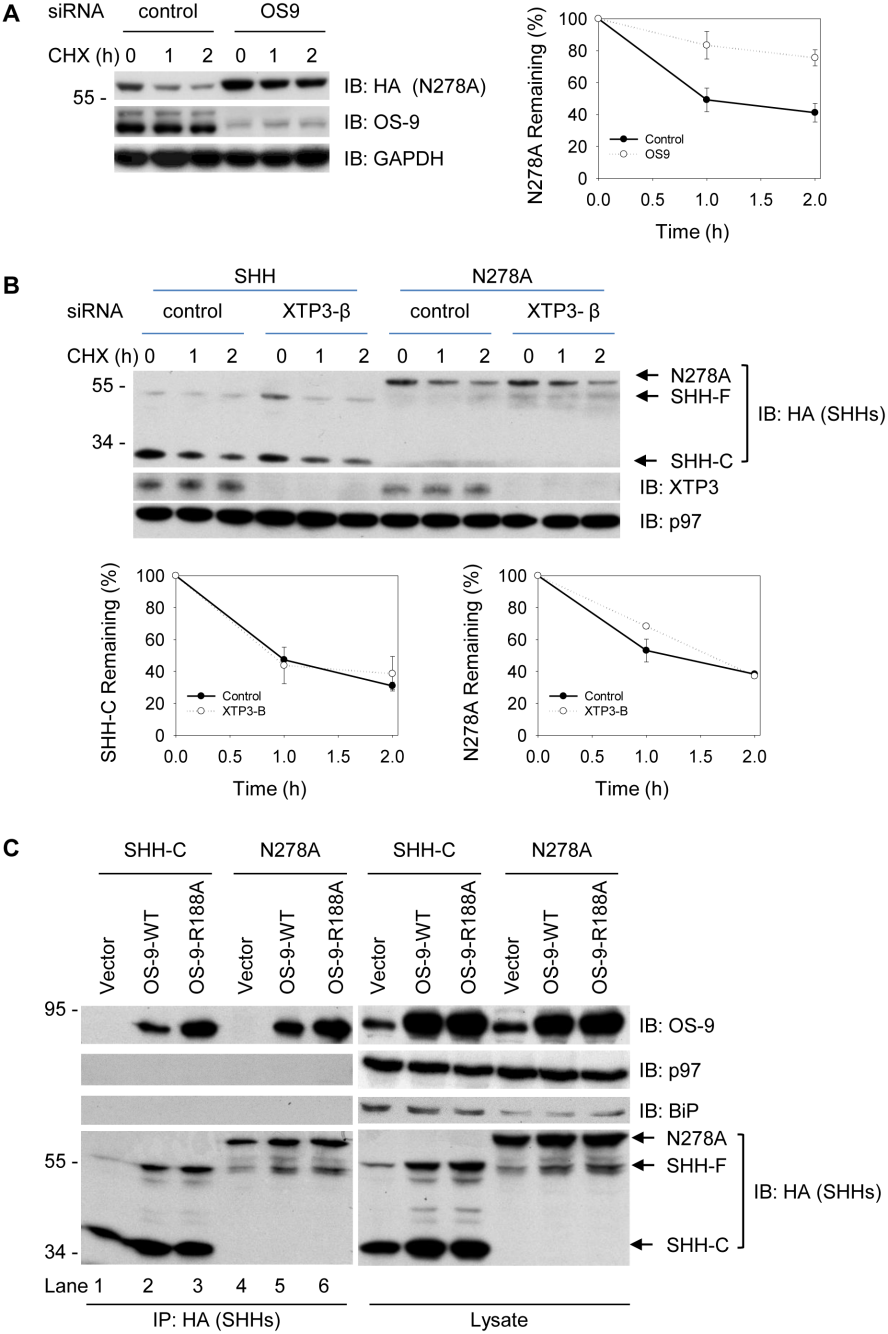
Contribution and interaction of OS-9 for degradation of SHH proteins. (**A**) Cells stably expressing the N278A-HA precursor were depleted of OS-9 by siRNA, and the fate of N278A was followed after cycloheximide (CHX) addition. All samples were analyzed by SDS-PAGE and immunoblotting with HA antibodies. Controls were treated with a commercial negative control siRNA. The right graph shows quantification of N278A from three independent experiments. The samples were also immunoblotted for OS-9 to show the extent of depletion. Immunoblotting with GAPDH antibodies served as loading control. (**B**) As in (A) but depletion of XTP3-B with cells stably expressing either SHH or N278A. (**C**) Plasmids expressing SHH-HA or N278A-HA were co-transfected with either vector, wild type OS-9.2 (WT-OS-9.2), or R188A-OS-9 mutant as indicated [30]. Cells were lysed in buffer containing 1% Triton and immunoprecipitated by HA antibodies. The precipitates and total cell lysate were separated in SDS-PAGE and immunoblotting with HA, p97, Bip and OS-9 antibodies.

Previously, we have shown that depletion of OS-9 is able to inhibit degradation of SHH-C [31]. To determine whether OS-9 and XTP3-B also participate in degradation of the unglycosylated N278A, we monitored N278A half-life in cells depleted of either lectin. While XTP-3 depletion had little on degradation of either SHH-C or N278A (Figure 6B), the loss of OS-9 markedly inhibited N278A degradation (Figure 6A), similar to its glycosylated counterpart. These data demonstrate a functional requirement for OS-9 (but not XTP3-B) in the degradation of SHH that does not depend on the substrate’s glycosylation state. In support of a role for OS-9 in their recognition, both SHH-C and N278A could be co-immunoprecipitated with the ER lectin (Figure 6C). Thus, it appears that the peptide backbone of SHH alone is sufficient for recognition by OS-9. As expected, the OS-9-SHH interaction did not require an intact MRH domain, as an OS-9-R188A mutant unable to bind trimmed glycans [25], [30] was co-precipitated by either SHH-C or N278A at levels even greater that wild type OS-9 (Figure 6C, lane 3 vs 2, 6 vs 5). Similar results have been reported previously for interactions between OS-9-R188A and the established ERAD substrate NHK [30]. These results indicate that an ERAD substrate’s interaction with OS-9 cannot be entirely explained by MRH domain-dependent glycan binding and support a model whereby the misfolded peptide segments play the determining role in recognition events. In this way, SHH proteins can be captured by either EDEMs or OS-9 through a misfolded polypeptide segment and in a glycan-independent manner.

### EDEMs Interact with Calnexin and SEL1L

**Figure 7:**
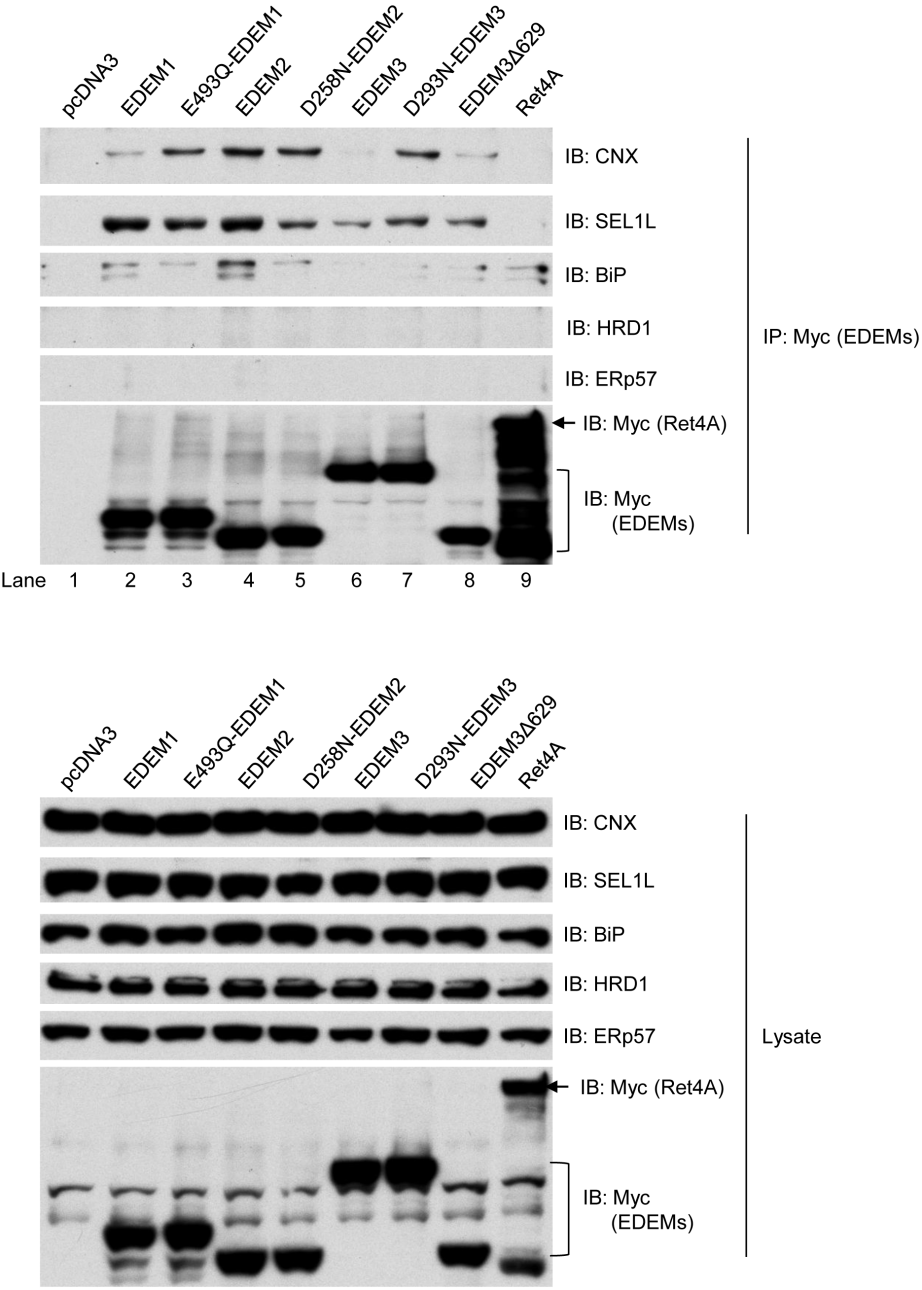
Interaction of EDEMs with calnexin and SEL1L. Plasmid expressing Myc-tagged EDEMs was transfected to 293T cells as indicated. Cells were lysed in buffer containing 1% Triton with protease inhibitors, and immunoprecipitated by Myc antibodies. As control, empty plasmid vector or plasmid encoding ER membrane protein Ret4A was transfected. The precipitates and total cell lysate were separated in SDS-PAGE and immunoblotting with Myc, SEL1L, HRD1, Bip, calnexin (CNX) and ERp57 antibodies.

The interaction between EDEM1 and calnexin can be detected when EDEM1 is transiently expressed in mammalian cells [34]. To ascertain whether other EDEM family members are also able to interact with calnexin, we transiently expressed EDEM1, 2 and 3 in 293T cells. Even at very low levels (10% of DNA normally used for transient transfection), we could reproducibly detect not only the EDEM1-calnexin interaction (Figure 7, lane 2) [34], but also much stronger interaction between EDEM2 and calnexin (Figure 7, lane 4). Compared to EDEM1 and EDEM2, the EDEM3-calnexin interaction was negligible (Figure 7, lane 6 vs 2 and 4). Notably, the calnexin cofactor ERp57 was not co-precipitated by any of the EDEM family, suggesting that ERp57 may be excluded from EDEM-calnexin complexes. We then asked whether their ability to bind glycans was important for EDEM1, 2 and 3 to bind calnexin. Both EDEM1_E493Q_ and EDEM3_D293A_ displayed increased association with calnexin (Figure 7, lane 3 vs 2, lane 7 vs 6), while EDEM2_D258N_ bound calnexin at levels equivalent to wild-type (Figure 7, lane 5 vs 4). These results suggest that interactions between the EDEM family and calnexin are not mediated through their respective glycan-binding domains.

EDEM1 and EDEM3, but not EDEM2, have been previously shown to interact with SEL1L [22], [35]. More specifically, the binding of EDEM1 to SEL1L is mediated by its glycan binding domain [22]. We could demonstrate that EDEMs differentially bind to SEL1L (Figure 7). EDEM1 and EDEM2 had the strong interaction with SEL1L, while EDEM3 bound SEL1L poorly (Figure 7, lanes 2, 4 and 6). Under relatively stringent buffer conditions (1% Triton X-100), neither HRD1 nor an unrelated ER membrane protein (Ret4A) was co-immunoprecipitated by EDEMs, arguing that the SEL1L-EDEM1-3 interactions detected were bona fide (Figure 7). In contrast to a previous report [35], we were able to detect an interaction between EDEM2 and SEL1L (Figure 7, lane 4). EDEM1_E493Q_ and EDEM2_D258N_ mutants consistently demonstrated reduced binding to SEL1L when compared to their wild type counterparts (lane 3 vs 2, 5 vs 4), in agreement with a previous report on EDEM1 interaction [22]. Similarly, EDEM3-D293N and Δ629 exhibited weaker interaction with SEL1L when compared to wt EDEM1 and EDEM2 (Figure 7, lanes 7 and 8 vs 2 and 4). As SEL1L is a confirmed glycoprotein, these results suggest that the glycan-binding domains of EDEM1/2 at least partially mediate the interaction with SEL1L, presumably via its glycans.

## Discussion

When encountering a misfolded substrate in the mammalian ER, how ER resident lectins recognize and target that substrate for ERAD is not yet fully understood. Mammals express three EDEM family members in the ER and their individual and collective functions remain only partially characterized. Here, we demonstrate that an unglycosylated form of SHH (N278A) is a robust ERAD substrate that requires essential ERAD components such as HRD1, SEL1L and p97 (Figure 1). Importantly, by systematically analyzing the requirement for EDEMs and ER lectins in degradation of SHH N278A, we could show that both glycosylated SHH-C and nonglycosylated N278A exhibit the greatest dependence on EDEM2 and OS-9 for ERAD (Figures 3, 4 and 7A), while EDEM3 involvement was limited to ERAD of glycosylated SHH-C (Figure 3). The lack of impact loss of either EDEM1 or XTP3-B suggests that neither plays a significant role in the ERAD of SHH-C or N278A. As there have been no previous reports implicating both OS-9 and EDEM2 in ERAD of a non-glycosylated substrate, our findings reflect the apparent versatility these lectins maintain in recognition and delivery of misfolded substrates.

As to the necessity of glycans for substrate recognition, we have provided evidence that such a signature is not absolutely essential for substrate recognition and delivery to SEL1L by EDEM2 and OS-9 (Figures 5 and 7). Rather, the deterministic signal for ERAD appears to be the misfolded polypeptide backbone. Not only are EDEM2 and OS-9 able to associate (either directly or indirectly) with non-glycosylated proteins, but they also play an essential role in targeting them for degradation. Our results reveal apparent differences in substrate recognition by the lectins in *S. cerevisiae* and their mammalian orthologs. In yeast, neither Htm1p nor Yos9p are involved in the ERAD of misfolded unglycosylated proteins [8], [9], [21]. The MRH glycan-binding domain of Yos9p is required for ERAD of glycoproteins but not for interaction [15]. To date, a mannosidase activity associated with EDEM2 has not been found [36]. It is noteworthy that the mutations at the presumed glycan-binding pockets have been used to probe the interaction of EDEM1 with other glycoproteins [22], even though it has not been definitely proven that the mutations indeed render the EDEM1 incapable of binding the glycoproteins. The mutant is presumed to eliminate the enzymatic activity as well as the glycan-binding abilities of EDEM1, which is largely extrapolated from the study for ER mannosidase I, and the sequence homology between EDEM1 and ER mannosidase I [22], [37]. It remains to be studied whether these presumed glycan-binding sites are indeed important for the function of EDEMs. Since we did observe an impact of EDEM2 loss on both SHH-C and N278A, it may be serving as an ER lectin/chaperone that is dedicated to the HRD1-mediated ERAD process. Furthermore, the robust interaction seen between EDEM2 and calnexin/SEL1L may be a way to ensure that misfolded glycoproteins are not released into the secretory pathway, but rather productively channeled from calnexin towards SEL1L for ERAD. In fact, the interactions of EDEM2 and calnexin are stronger than either EDEM1 or EDEM3. It is noteworthy that EDEM2 does not have a KDEL sequence for ER retention [29], [38] and as such, may also rely on its interaction with either SEL1L or/and calnexin to anchor it in ER.

A recent study implicated EDEM3 in the degradation of glycosylated TTR mutant proteins [39]. But even though mannosidase activity for EDEM3 been shown *in* *vivo* [36], it is still not clear whether mannose processing by EDEM3 was essential for degradation of the mutant proteins [39]. And even though EDEM3 contributed to the degradation of glycosylated SHH-C, it may not be utilizing the SHH-C glycan for its recognition and degradation. This argument is based on the following observations. First, over-expression of EDEM3 active site mutants failed to impair SHH degradation (Figure S3), suggesting that enzymatic processing by EDEM3 of SHH-C might not be essential. Second, interactions with EDEM3/EDEM2 occurred independently of the ERAD substrates’ glycosylation status (Figure 5). Finally, OS-9 interaction with misfolded SHH proteins also did not require the MRH domain and occurred independently of the substrates glycans (Figure 6C). This implies that mannose processing is dispensable for substrate binding to OS-9, at least in the case of SHH. The mechanistic action of EDEM3 in mediating degradation of SHH-C may differ from that of NHK proteins, in which mannose processing occurs and is required [28]. When compared to EDEM1 or EDEM2, EDEM3 exhibited the weakest interaction with either calnexin or SEL1L, indicating that it may operate in a manner significantly different from that of either EDEM1 or 2. Even though EDEM1 and EDEM3 do not contribute to the degradation of non-glycosylated N278A proteins, they retain the ability to interact with non-glycosylated proteins (Figure 5). The binding of lectins to the misfolded substrates without directly contributing to their degradation has been observed in many instances before [22], [24], [28], [29], [30].

In summary, our studies have demonstrated that EDEM2 and OS-9 contribute to the degradation of misfolded proteins, independently of the substrates’ glycosylation status. We found that both EDEM2 and EDEM3 participate in the degradation of glycosylated SHH-C and that substrate recognition occurs independently of either the substrate glycan or the lectin-like domain. This study reveals distinct properties in the mammalian ER lectins that differentiate them from their counterparts in *S. cerevisiae*, and sheds light on the diverse roles played by these lectins in mammalian ERAD. Further investigations will be necessary to fully ascertain the differences these ER lectins impart on the mammalian ERAD mechanism.

## Materials and Methods

### Materials

The materials used in this study have been described previously [40] except the following: Anti-Myc and anti-RGS-His antibodies were purchased from Roche, ERp57 and calreticulin antibodies from GeneTex Inc., Taiwan, XTP3-B antibodies from Santa Cruz, anti-OS-9 antibodies from Abcam (Novus), and anti-EDEM1 and EDEM3 antibodies from Sigma. CST and KIF were purchased from Enzo Life Science and Cayman Chemical Company, respectively.

### DNA Constructs for Mammalian Cell Transfection

Full length SHH with HA tag in pIRES2-EGFP (Clontech) was described previously [31]. Expression plasmids coding for EDEM1, EDEM2 and EDEM3 were generated by PCR from human cDNA libraries, and cloned to pIRES2-EGFP vector. Site-directed mutagenesis was carried out using the QuikChange kit (Strategen) to generate N278A on SHH or EDEMs’ mutations used in the study. Expression plasmids for OS-9.2 and XTP3-B are described in detail elsewhere [30]. Wild type and catalytically inactive p97 (p97-wt and p97-QQ) and HRD1 (HED1-WT and HRD1-C291A) were provided by Y. Ye (National Institutes of Health, Bethesda, MD, USA) and E. Wiertz (Leiden University, Leiden, Netherlands), respectively. Myc-tagged reticulon 4A was obtained from S.M. Strittmatter (Yale University, CT).

### Cell Culture and Generation of Stable Cell Lines

Human 293T cells were grown in DMEM (Invitrogen) supplemented with 10% fetal bovine serum, penicillin and streptomycin. To generate stable cell lines, constructs encoding full length SHH-HA or N278A-HA in pIRES2-EGFP vector were transfected to 293T cells and selected for cells expressing the proteins by flow cytometry. The expression of the SHH proteins were confirmed by western blot analysis with HA antibodies.

### Cycloheximide Chase Assays

Cycloheximide chase assay was carried out as described [31]. Cells were cultured in 6-well plates, incubated with 100 ug/ml cycloheximide in culture medium at 37°C. At the times indicated in the figures, cells were removed and lysed on ice for 10 mins in buffer containing 50 mM Tris-HCl, pH 7.4, 150 mM NaCl and 1 mM EDTA supplemented with protease inhibitors and 1% Triton. The lysate was centrifuged for 20 mins at 4°C and at 20,000 g. The supernatant was collected, mixed with SDS-PAGE sample buffer with β-mercaptoethanol (2% final concentration) before immunoblotting.

### Transfection of Plasmids and siRNAs

Transfection procedures have been described previously [31], [40]. Specifically, one tenth of the usual amount of DNA (0.3 ug of the SHH expression plasmids and 0.9 ug of the lectin expression plasmids) were used to transfect one 10 cm plate in order to minimize the overexpression effect. siRNA sequences used are shown in Table S1.

### Quantitative RT-PCR Analysis

The procedure has been described previously [31], [40]. The primers used to quantify mRNA knockdown are shown in [31] and Table S2.

### Immunoblotting and Immunoprecipitation

The procedure has been described [31], [40]. Immunoprecipitation was carried out either in HKM buffer (25 mM HEPES, pH 7.5, 100 mM KOAc, 2 mM MgOAc) with 1% digitonin, or buffer containing 20 mM Tris-HCl, pH 7.4, 135 mM NaCl, 1% Triton and 10% glycerol, all supplemented with protease inhibitors (Roche).

## Supporting Information

Figure S1 **N278A proteins are not glycosylated.** Cell lysate were prepared from the cells stably expressing either N278A-HA (lanes 1 and 2) or SHH-HA precursor (lanes 3 and 4), treated with PNGase. The lysate were separated on SDS-PAGE and immunoblotting with HA antibodies. The faster migrating bands indicated were the de-glycosylated full length SHH (SHH-F) and SHH-C proteins. Immunoblotting for p97 serves as loading control.
(TIF)

Figure S2 **Enzyme dead mutants of HRD1 and p97 inhibit N278A degradation. (A)** Cells stably expressing the N278A were transfected with a catalytically inactive Myc-tagged HRD1 (HRD1-C291A) or with an empty vector. The fate of HShh-HA was followed after addition of cycloheximide (CHX). All samples were analyzed by SDS-PAGE followed by immunoblotting for Myc (HRD1-C291A) and HA. Immunoblotting for p97 served as a loading control. The right graph shows the quantification of N278A in the experiment. **(B)** As in A, but with transfection of either wild type p97 (p97-wt), a catalytically inactive p97 mutant (p97-QQ), or with an empty vector. p97 were detected by immunoblotting with p97 and His antibodies. Immunoblotting for GAPDH served as a loading control. The lower graph shows the quantification of N278A in the experiment.
(TIF)

Figure S3 **Overexpression of wt and mutant EDEM3 has no effect on the ERAD of SHH-C.** Cells stably expressing the SHH-HA precursor were transfected with either Myc-tagged wild type EDEM3 or its catalytic mutant EDEM3_D293N_ [22]. The fate of SHH-HA was followed after addition of cycloheximide (CHX), by SDS-PAGE and immunoblotting for HA and EDEM3 antibodies. Immunoblotting for actin served as a loading control. The lower graph shows the quantification of the SHH-C in the experiment.
(TIF)

Table S1 **Sequences of siRNA duplexes used in this study.**
(DOCX)

Table S2 **Primer sequences for quantitative PCR used in this study.**
(DOCX)
