# Supplementary figures and images for: EDEM2 and OS-9 Are Required for ER-Associated Degradation of Non-Glycosylated Sonic Hedgehog
Source: PLoS One. 2014 Jun 9;9(6):e92164. doi: 10.1371/journal.pone.0092164 (PMC4049591; doi:10.1371/journal.pone.0092164)

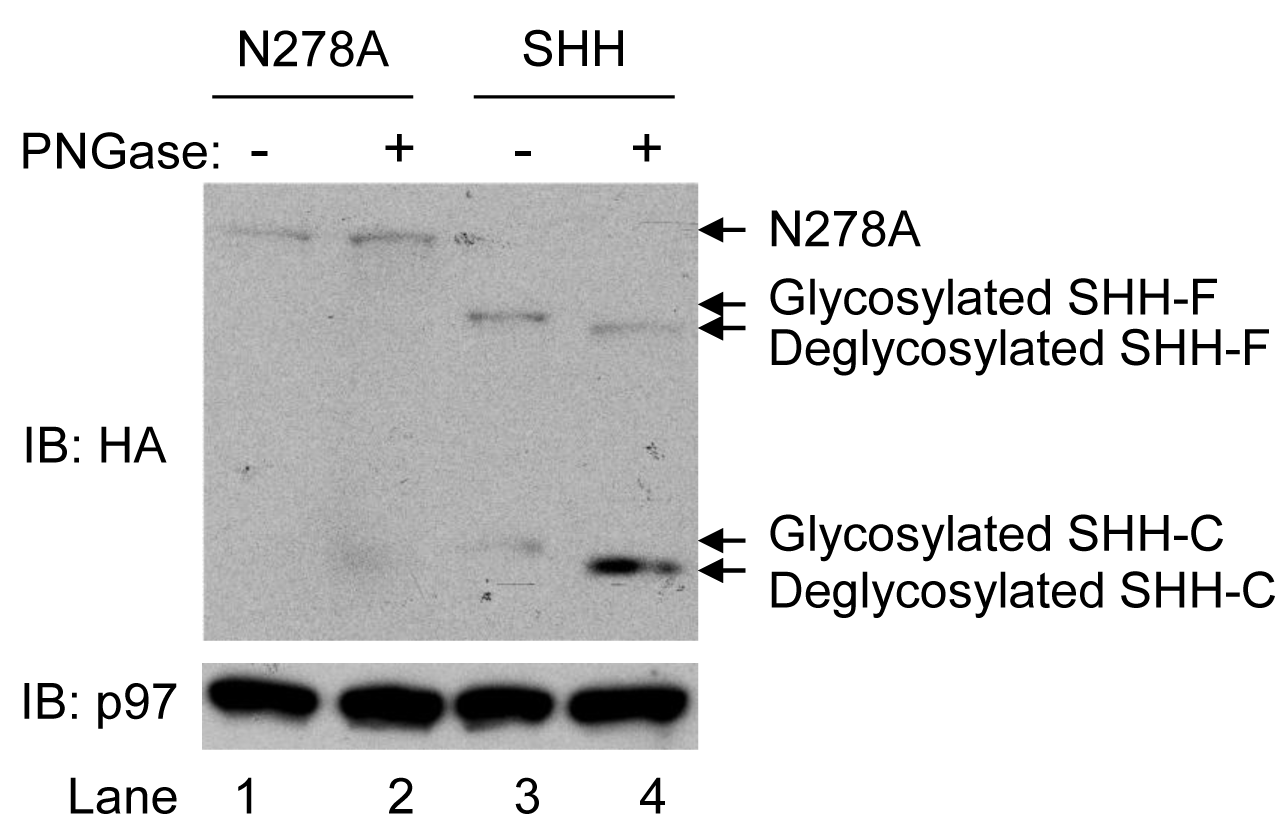

Supplement: Figure S1 — N278A proteins are not glycosylated. Cell lysate were prepared from the cells stably expressing either N278A-HA (lanes 1 and 2) or SHH-HA precursor (lanes 3 and 4), treated with PNGase. The lysate were separated on SDS-PAGE and immunoblotting with HA antibodies. The faster migrating bands indicated were the de-glycosylated full length SHH (SHH-F) and SHH-C proteins. Immunoblotting for p97 serves as loading control. (TIF) [file pone.0092164.s001.tif]

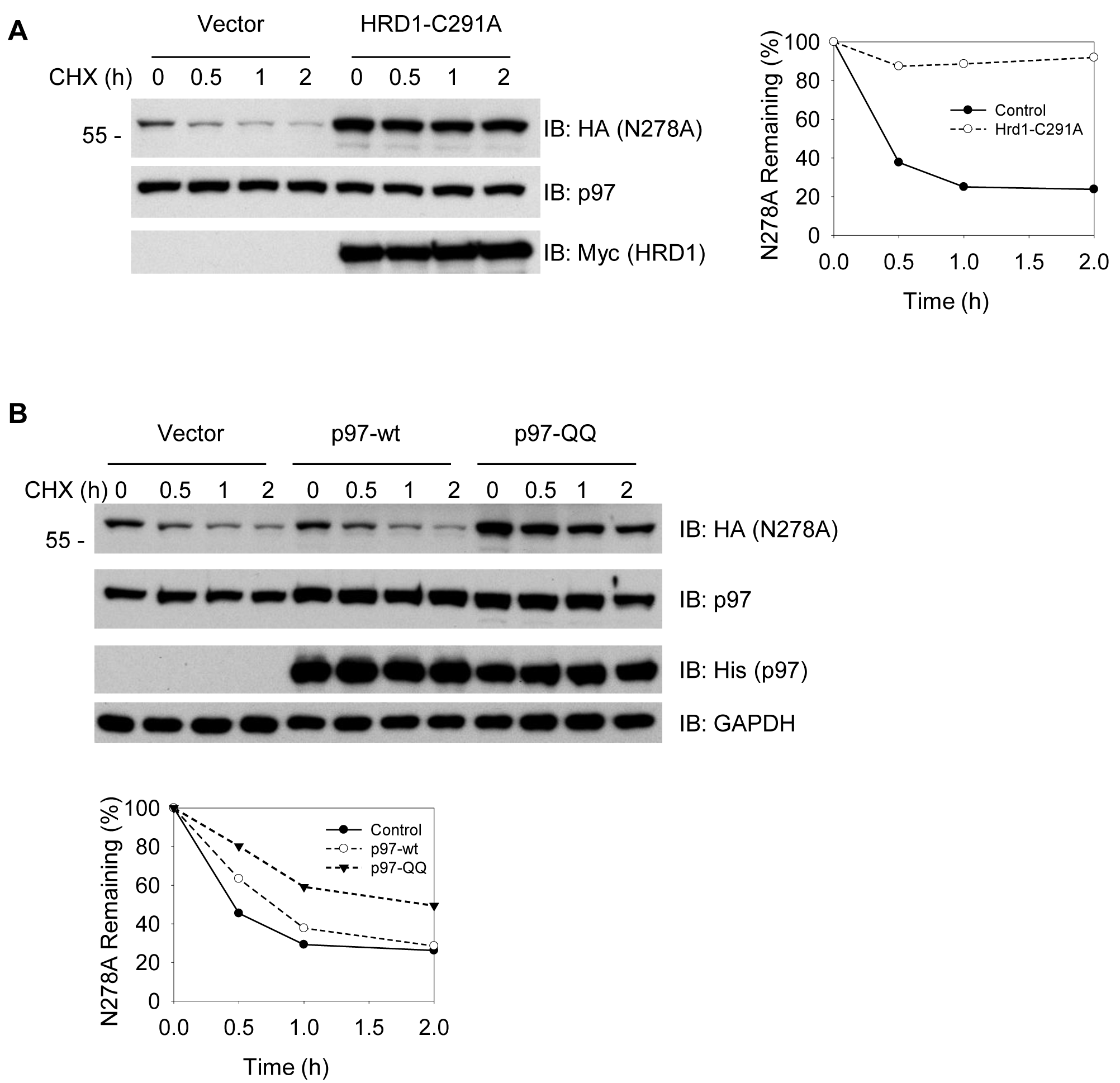

Supplement: Figure S2 — Enzyme dead mutants of HRD1 and p97 inhibit N278A degradation. (A) Cells stably expressing the N278A were transfected with a catalytically inactive Myc-tagged HRD1 (HRD1-C291A) or with an empty vector. The fate of HShh-HA was followed after addition of cycloheximide (CHX). All samples were analyzed by SDS-PAGE followed by immunoblotting for Myc (HRD1-C291A) and HA. Immunoblotting for p97 served as a loading control. The right graph shows the quantification of N278A in the experiment. (B) As in A, but with transfection of either wild type p97 (p97-wt), a catalytically inactive p97 mutant (p97-QQ), or with an empty vector. p97 were detected by immunoblotting with p97 and His antibodies. Immunoblotting for GAPDH served as a loading control. The lower graph shows the quantification of N278A in the experiment. (TIF) [file pone.0092164.s002.tif]

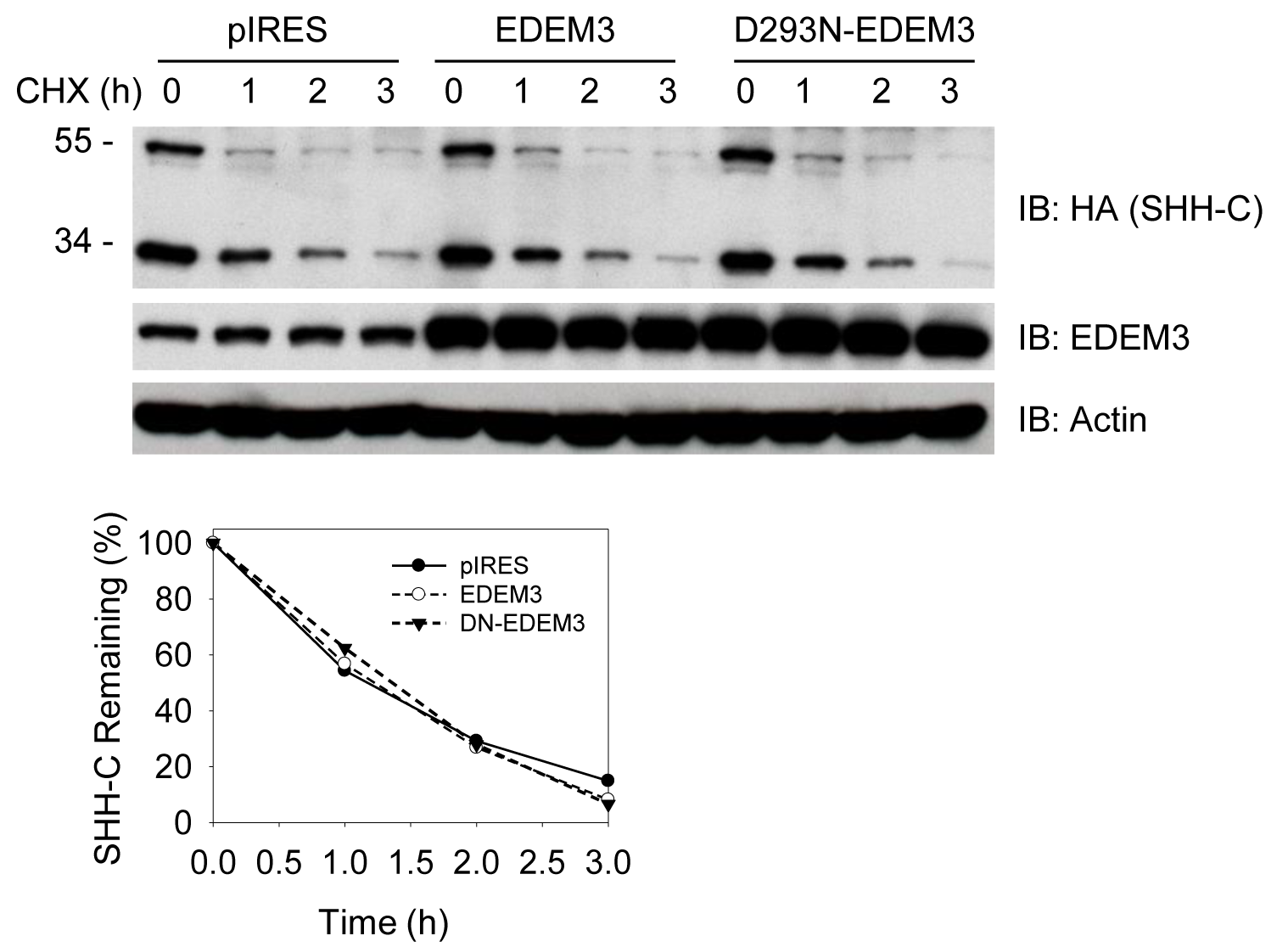

Supplement: Figure S3 — Overexpression of wt and mutant EDEM3 has no effect on the ERAD of SHH-C. Cells stably expressing the SHH-HA precursor were transfected with either Myc-tagged wild type EDEM3 or its catalytic mutant EDEM3D293N [22]. The fate of SHH-HA was followed after addition of cycloheximide (CHX), by SDS-PAGE and immunoblotting for HA and EDEM3 antibodies. Immunoblotting for actin served as a loading control. The lower graph shows the quantification of the SHH-C in the experiment. (TIF) [file pone.0092164.s003.tif]
