# Supplementary material for: EDEM2 and OS-9 Are Required for ER-Associated Degradation of Non-Glycosylated Sonic Hedgehog
Source: PLoS One. 2014 Jun 9;9(6):e92164. doi: 10.1371/journal.pone.0092164 (PMC4049591; doi:10.1371/journal.pone.0092164)
Supplement: Table S1 — Sequences of siRNA duplexes used in this study. (DOCX) [file pone.0092164.s004.docx]

**Supplemental Table S1: Sequences of siRNA duplexes used in this study.**

| siRNA | Nucleotides | Primers (forward and reverse) |
| --- | --- | --- |
| EDEM1-A | 792 | CGUCCAAGUCUUUGAGGCCACGAUA  UAUCGUGGCCUCAAAGACUUGGACG |
| EDEM1-B | 1149 | UAGUAAUCCUGUAUCAUUGCUCCGG  CCGGAGCAAUGAUACAGGAUUACUA |
| EDEM2-A | 872 | UCACCAAGUACUCAAAGUAGGAGUC  GACUCCUACUUUGAGUACUUGGUGA |
| EDEM2-B | 1280 | AGAGAUGCUGUGGAAUCCAUUGAAA  UUUCAAUGGAUUCCACAGCAUCUCU |
| EDEM3-A | 1741 | CAUCUGUUACCUCUUUGGCUCUCUA  UGGUCCAGCAGUCAAUACUACCCUG |
| EDEM3-B | 2238 | UGGUCCAGCAGUCAAUACUACCCUG  CAGGGUAGUAUUGACUGCUGGACCA |
| XTP3-β | 1597 | AGAUUCACCUCAUGCUGUUACUGUA  UACAGUAACAGCAUGAGGUGAAUCU |
