# Supplementary material for: EDEM2 and OS-9 Are Required for ER-Associated Degradation of Non-Glycosylated Sonic Hedgehog
Source: PLoS One. 2014 Jun 9;9(6):e92164. doi: 10.1371/journal.pone.0092164 (PMC4049591; doi:10.1371/journal.pone.0092164)
Supplement: Table S2 — Primer sequences for quantitative PCR used in this study. (DOCX) [file pone.0092164.s005.docx]

**Supplemental Table S2: Primer sequences for quantitative PCR used in this study**

| [*Homo sapiens*](http://www.ncbi.nlm.nih.gov/nucest?term=NM+0012763##)gene | Nucleotides | Primers (forward and reverse) |
| --- | --- | --- |
| EDEM1 | 1399-1609 | AAACGATATGGTGCCCTCCCTG  CGTGATGCAGCGTGGCGTAC |
| EDEM2 | 244-420 | CTGGACACCTTGCTGATTTTGG  TACTTCCACCCCAGCCTTCTTG |
| EDEM3 | 856-1031 | AGTGGAGTTGGAGCAGGGATTG  GCATTCAGCATTGGTTTGTGGA |
| OS-9 | 247-396 | CAGCGTGAAAGGGAGGAGGAAA  GTGGTATTGCTGGATGTGGCGT |
| XTP3-B | 254-406 | CAAGTGGGGATGAGGAAGAAGA  CATGGTACTGCCGAATGTGTTT |
